# Supplementary material for: Arabidopsis thaliana SHOOT MERISTEMLESS Substitutes for Medicago truncatula SINGLE LEAFLET1 to Form Complex Leaves and Petals
Source: Int J Mol Sci. 2022 Nov 15;23(22):14114. doi: 10.3390/ijms232214114 (PMC9697493; doi:10.3390/ijms232214114)
Supplement: Supplementary file 1 [file ijms-23-14114-s001.zip › ijms-2000059-supplementary.pdf]

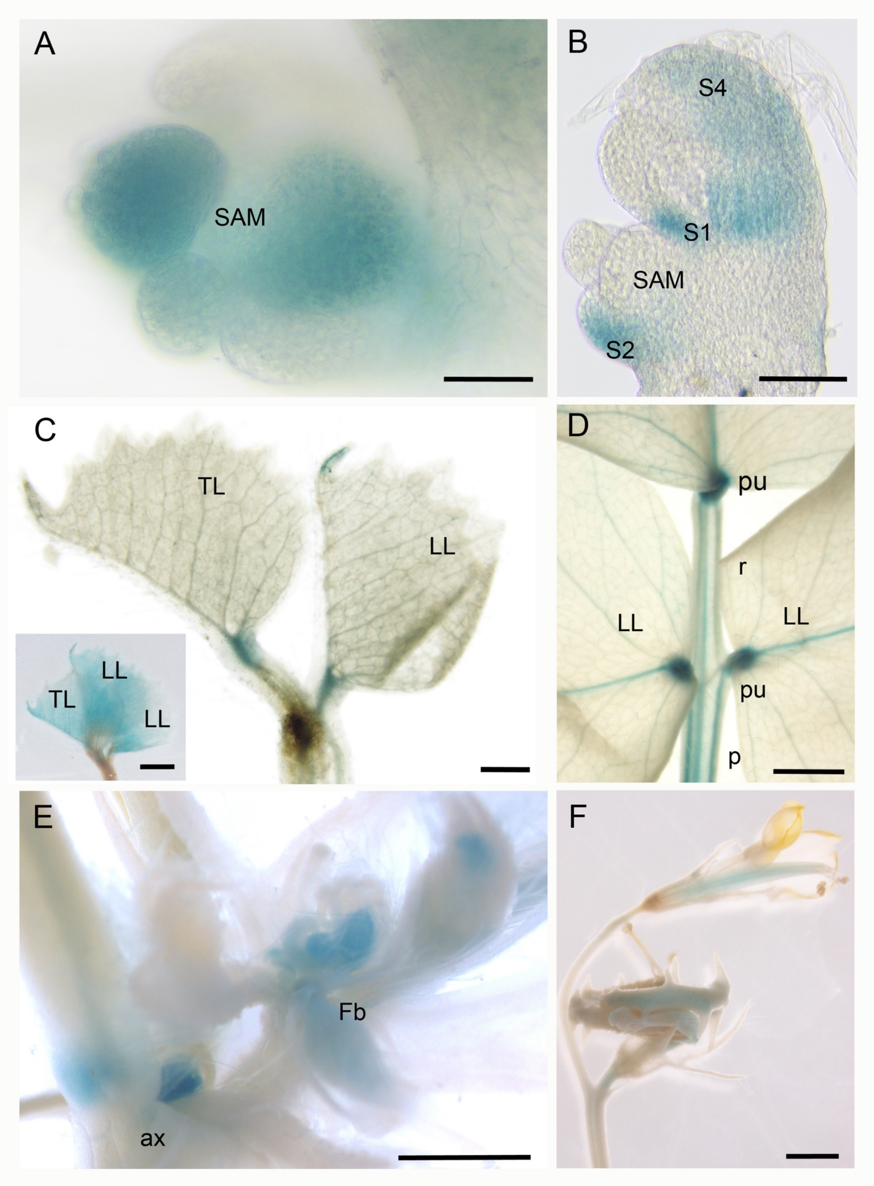

Figure S1. *pSGL1*:*GUS* expression in *M. truncatula*. (A) and (B) *pSGL1:GUS* expression in vegetative apices. *pSGL1:GUS* expression was detected in developing S2 and S4 leaf primordia, (C) inset: *pSGL1:GUS* expression in young leaves. At later stages, *pSGL1:GUS* expression was detected at the basal region of terminal (TL) and lateral (LL) leaflets in the pulvinus (pu), (D) Close-up view of the basal region of leaflets showing *pSGL1:GUS* expression in the pulvinus (pu) and in vascular tissues of petiole (p) and rachis (r), (E) *pSGL1:GUS* expression in reproductive apices. *pSGL1:GUS* expression was detected in young developing floral buds (Fb) and in axillary meristem (ax), (F) *pSGL1:GUS* expression in carpels and fruits. Bars: A,B = 50 μm, C inset, E, F = 1 mm, C,D = 2 mm.

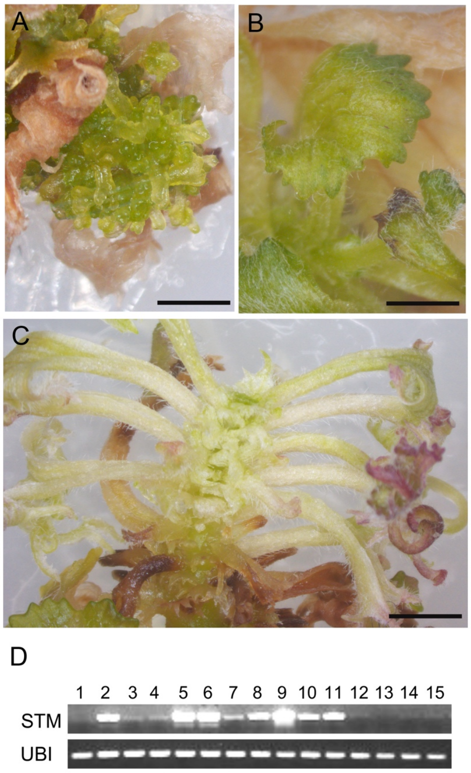

Figure S2. *In vitro* transgenic *M. truncatula* plantlets overexpressing *AtSTM*. (A-C) *In vitro p35S:AtSTM* transgenic *M. truncatula* plantlets showing a severe phenotype. These plants did not survive when transferred into greenhouse, (D) Levels of *AtSTM* transcript in wild type *M. truncatula* control R108 plant (Lane 1) and transgenic lines expressing *p35S:AtSTM* (lanes 2-15). Lines that express *AtSTM* at high levels (lanes 2, 5, 6, 8, 9, 10 and 11) show a severe phenotype and did not survive after transfer into greenhouse. Lines expressing *AtSTMs* at low levels were further characterized. Primers specific for the *M. truncatula UBIQUITIN* gene were used as an internal control. Bars = 0.5 cm.

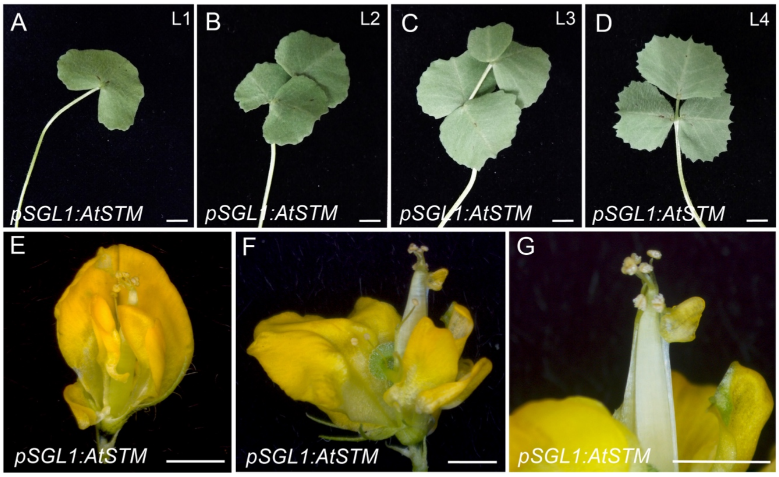

Figure S3. Phenotype of a *pSGL1:AtSTM* transgenic line expressing *AtSTM* under the *pSGL1* promoter. (A-D) Phenotype of juvenile (L1) and adult leaves (rank L2-L4) of 5-week-old plants, showing a L3 leaf with 4 leaflets. This phenotype was occasionally observed, (E-G) Phenotype of *pSGL1:AtSTM* flowers, showing dissected petals (E) and petal sectors on stamens (F-G). Bars A-D = 5 mm, E-G = 2 mm.

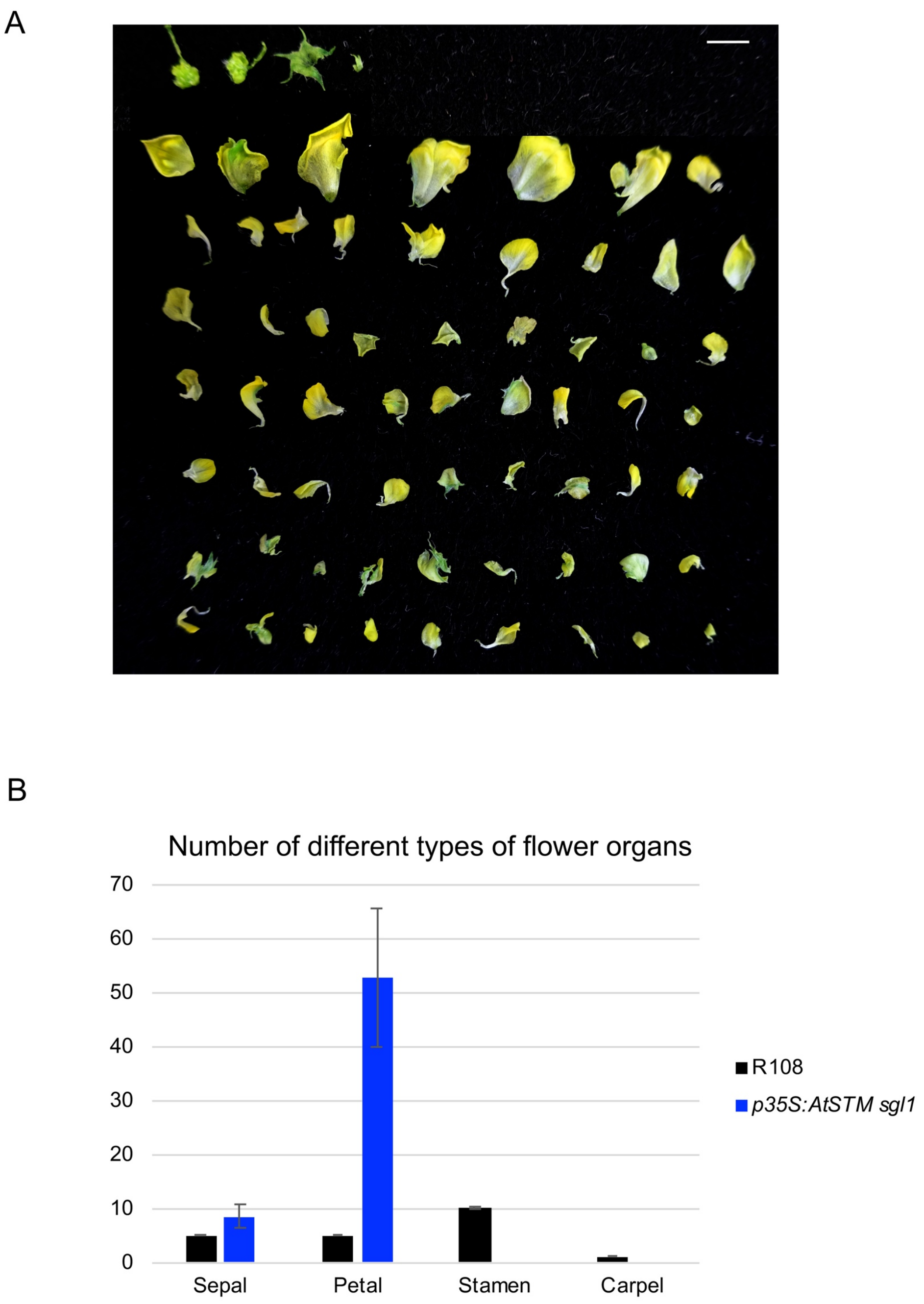

Figure S4. Petals production following *AtSTM* expression in *sgl1* flowers. (A) A dissected *p35S:AtSTM sgl1* flower, comprising a calyx with 5 sepals, 2 sepals 61 petals, some of which showing sepal sectors and incomplete floral meristems with unfully differentiated petals and sepals. Bars = 5 mm, (B) Quantification of floral organs number. Average ± SD (n=20) of sepals, petals, stamens and carpels in wild type (R108) and in *p35S:AtSTM sgl1* flowers. Petals in these flowers represent petals (80%) and petals with sepal sectors (20%).

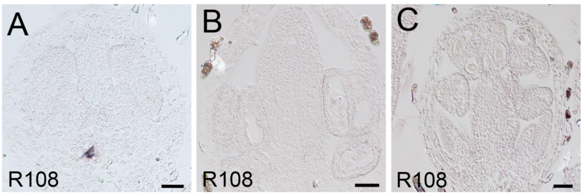

Figure S5. *In situ* hybridization with control sense probes. (A) *MtAP1* sense control in wild-type flower, showing no signal, (B) *MtPI* sense control in wild-type flower, showing no signal, (C) *MtAgb* sense control in wild-type flower, showing no signal. Bars = 50 μm.

Table S1. List of primers

primer	Sequence 5'-3'
**Genotyping**	
SGL1-for	GCTTACCATGGATCCCGACGCATT
SGL1-rev	TAACTTAAAAAGGAAGGTGAGCAGTTC
Tnt1-upstream	CTCCAGACATTTTTATTTTTCACCAAG
Tnt1-downstream	GCATTCAAACTAGAAGACAGTGCTACC
**Transgenics**	restriction site is underlined
pSGL1-for	TAGACAAGATCTCAAAAATGGTGTACCAAA
pSGL1-rev	CGTCGGGATCCATGGTAAGCAATG
pAtSTM-for	GGGGATCCGACTCTAGTAAAA
pAtSTM-rev	GGGAATTCAATTGCCATGTCA
**RT-PCR**	
qSTM-F	GGTGGAGCCGTCACTACAAA
qSTM-R	ATGGTGAGG ATGTGTTGCGT
qUBI-F	GCAGATAGACACGCTGGG
qUBI-R	AACTCTTGGGCAGGCAATAA
**In *situ* hybridization**	
MtPIM_ISH.Fw	GTATACGCGACTGAAGGCAAAG
MtPIM_ISH.Rev	TGGCAGGTATACAATGGTTCC
MtPI_ISH.Fw	GGCACTTGAAGGTGTGGGAAA
MtPI_ISH.Rev	GAAACCAAATTCAATCACTTCATA
MtAGb_ISH.Fw	GATATCAGAAAGTGAGCAGAG
MtAGb_ISH.Rev	GTCTTTGCTCTTCTCAACCG
